# 2108 Factors associated with dual use of VA and civilian healthcare among U.S. National Guard and Reserve soldiers

**DOI:** 10.1017/cts.2018.285

**Published:** 2018-11-21

**Authors:** Bonnie M. Vest, Jessica A. Kulak, D. Lynn Homish, Gregory G. Homish

**Affiliations:** 1 University at Buffalo, State University of New York; 2 Department of Family Medicine, University at Buffalo, State University of New York; 3 Department of Community Health and Health Behavior, University at Buffalo, State University of New York

## Abstract

OBJECTIVES/SPECIFIC AIMS: Approximately 25%–45% of veterans are dual users of VA and civilian healthcare. In order to maximize patient outcomes, understanding factors related to dual use is important. This study examined mental and physical health factors related to dual use of VA and civilian healthcare among U.S. National Guard and Reserve (NG/R) soldiers. METHODS/STUDY POPULATION: NG/R soldiers and their partners (n=411 couples) participated in an electronic survey assessing health and health behaviors. Logistic regression models were used to examine the relationship between mental health (anxiety, depression, PTSD, anger), general health, and VA disability status at baseline, with usage of both VA and civilian healthcare among male soldiers (n=109) at the second year follow-up, controlling for age and race. RESULTS/ANTICIPATED RESULTS: In the final adjusted models, of the mental health conditions, only anxiety was related to dual use (OR: 1.08, 1.01–1.16, *p*<0.05). Having a VA disability rating (OR: 4.00, 1.22–13.18; *p*<0.05) was also related to being a dual user. General health was not related to dual use. DISCUSSION/SIGNIFICANCE OF IMPACT: While research has identified demographic characteristics (e.g., rurality, race, income) related to dual healthcare use, our results indicate that mental health, particularly anxiety, may also be related to dual use. Further study is needed to tease out the prime drivers of dual use to identify future care delivery mechanisms that will maximize treatment outcomes and minimize duplicative care.

